# Effect of Inflammation on Female Gonadotropin-Releasing Hormone (GnRH) Neurons: Mechanisms and Consequences

**DOI:** 10.3390/ijms21020529

**Published:** 2020-01-14

**Authors:** Klaudia Barabás, Edina Szabó-Meleg, István M. Ábrahám

**Affiliations:** 1 Molecular Neuroendocrinology Research Group, Institute of Physiology, Medical School, Centre for Neuroscience, Szentágothai Research Institute, University of Pécs, H-7624 Pécs, Hungary; klaudia.barabas@aok.pte.hu; 2 Departement of Biophysics, Medical School, University of Pécs, H-7624 Pécs, Hungary; edina.meleg@aok.pte.hu

**Keywords:** GnRH neuron, estradiol, inflammation, cytokines, obesity

## Abstract

Inflammation has a well-known suppressive effect on fertility. The function of gonadotropin-releasing hormone (GnRH) neurons, the central regulator of fertility is substantially altered during inflammation in females. In our review we discuss the latest results on how the function of GnRH neurons is modified by inflammation in females. We first address the various effects of inflammation on GnRH neurons and their functional consequences. Second, we survey the possible mechanisms underlying the inflammation-induced actions on GnRH neurons. The role of several factors will be discerned in transmitting inflammatory signals to the GnRH neurons: cytokines, kisspeptin, RFamide-related peptides, estradiol and the anti-inflammatory cholinergic pathway. Since aging and obesity are both characterized by reproductive decline our review also focuses on the mechanisms and pathophysiological consequences of the impact of inflammation on GnRH neurons in aging and obesity.

## 1. Introduction

The hypothalamic–pituitary–gonadal axis (HPG axis) regulates reproduction. Gonadotropin-releasing hormone (GnRH) neurons are the central regulators of fertility. They are small, fusiform cells scattered throughout the hypothalamus and basal forebrain (medial septum (MS) preoptic area (POA), with fibers projecting to the median eminence (ME) and the organum vasculosum of the laminae terminalis (OVLT) [1]. GnRH is a decapeptide that acts on the anterior pituitary (AP) to control the production and release of follicle-stimulating hormone (FSH) and luteinizing hormone (LH), which regulate gonads: Testosterone production from testes and estradiol and progesterone from ovaries.

GnRH secretion is finely governed by excitatory and inhibitory transsynaptic neuronal inputs. Kisspeptin, a KISS-1 gene product was identified as the main regulator of episodic GnRH release. Kisspeptin is a neuropeptide expressed predominantly in the rostral periventricular area of the third ventricle (RP3V) and arcuate nucleus (ARC) in rodents [2] or in the RP3V and infundibular nucleus (equivalent to the rodent ARC) in humans [3]. In addition, the role of two other neuropeptides has been described in GnRH pulse generation, neurokinin B (NKB) and dynorphin. They have been demonstrated to co-localized with kisspeptin in the arcuate nucleus creating the kisspeptin/neurokinin B/dynorphin (KNDy) neurons [4]. According to the “KNDy hypothesis” NKB initiates the pulse onset, kisspeptin is the output signal to drive GnRH secretion and finally, dynorphin serves as an inhibitory signal to terminate the pulse [5]. Morphological studies showed that KNDY neurons are connected with each other via axo-somatic synapses [4].

In addition to kisspeptin, gonadotropin inhibitory hormone (GnIH) is a lately discovered neuropeptide in birds that regulates the HPG axis in physiological conditions [6]. Similarly, mammalian GnIH orthologs, known as RFamide-related peptides (RFRPs) suppress the function of HPG axis. GPR147, the receptor of RFP is expressed in the hypothalamus and pituitary as well and the RFamide-related peptide-3 (RFRP3) has been shown to act on GnRH neurons in the hypothalamus and also on the pituitary to inhibit GnRH and LH release and synthesis, respectively [7]. Besides that RFRP-3 neurons regulate GnRH and pituitary neurons, they also influence LH secretion acting on kisspeptin neurons [8]. However, the effect of RFRP-3-induced actions on kisspeptin neurons is controversial and are species- and sex-dependent [9,10,11].

Estradiol has a critical regulatory effect upon the activity of GnRH neurons in females that is indispensable for normal reproductive functions. During the estrous cycle, GnRH is secreted in a pulsatile manner, which is mainly controlled by the negative feedback actions of estradiol secreted from the ovaries [12]. In the preovulatory stage, GnRH is secreted in a surge induced by the positive feedback effects of estradiol released from the mature ovarian follicles finally evoking LH surge and consequently ovulation [13,14]. The positive feedback effects of estradiol on GnRH neurons occur through kisspeptin neurons that project to the cell body and proximal dendrites of GnRH neurons [1]. Although the critical role of intracellular signaling molecules such as cAMP responsive element binding protein has been proposed in estradiol-induced negative feedback action on GnRH neuron the precise mechanism remains elusive [15].

Besides its well-known role in fertility, the HPG axis acts in concert with the immune system to control immune functions. The relationship between the immune system and the HPG axis is bidirectional: Gonadal hormones have an impact on the immune system, but alterations in the immune function can elicit functional modifications of the HPG axis as well.

The interaction between the immune system and the HPG axis is primarily based on their shared receptors and mediators [16]. Primary substances that mediate signals from the immune system to GnRH neurons are the cytokines such as IL-1, TNF-α, and IL-10. Cytokines are essential in maintaining homeostasis and for regulating immune responses in the brain. The unbalanced production of pro- and anti-inflammatory cytokines has been linked to the progression of various human neurological disorders. Inflammation of the central nervous system (CNS) is now associated with nearly all neurological diseases. Neuroinflammation develops via peripheral immune cells migrating into the CNS [17] or local cytokine synthesis in the brain parenchyma [18]. Some amounts of blood borne cytokines can also cross the blood–brain barrier (BBB) with a saturated transport mechanism [19].

As there have been compelling studies published recently about the functional relevance of inflammation affecting the function of GnRH neurons, our review will focus on the mechanisms and the effect of inflammation on the GnRH neurons. We will discuss the neuroinflammatory processes and the effects of inflammation on fertility. As part of the mechanism, the role of cytokines, kisspeptin, RFamide-related peptides, estradiol, and the cholinergic pathway in inflammation will be reviewed. In regard to consequences, clinical aspects will be surveyed with special attention given to the neuroinflammatory processes in aging and the effect of obesity-induced inflammation on reproductive functions.

## 2. Neuroinflammatory Processes: The Role of Blood–Brain Barrier (BBB), Astrocytes, and Microglia

In order to understand the effects of neuroinflammation on GnRH neurons we first discuss the role of key players in the development of neuroinflammation such as BBB, astrocytes, and microglia.

BBB restricts the passage of large molecules from the bloodstream into the brain by forming tight endothelial junctions. Under physiological conditions the access of peripheral immune cells into the CNS is prevented by the BBB. When bacterial or viral infections disrupt the BBB, circulating immune cells, such as lymphocytes (B and T cells), monocytes and granulocytes can enter the brain parenchyma supplying a source of cytokines [20,21,22,23]. Additionally, cytokines can be secreted locally in the brain parenchyma [18], the BBB [24] and choroid plexus cells [25] during peripheral inflammation initiating inflammatory processes in the CNS.

Another possible way for cytokines to enter the brain is through the circumventricular organs. The circumventricular organs such as OVLT and ME are specialized sites lacking the BBB that allows direct communication between the blood and brain parenchyma. It has been established that most GnRH neurons send axons to the external zone of the median eminence to secrete GnRH in a pulsatile manner into the pituitary portal system to control gonadotropin release. However, the detailed structure and function of a very high density of GnRH fibers found within the OVLT has remained unexplored until recently. Herbison and his colleagues reported that the cell bodies of GnRH neurons located within 100 μm of the OVLT extend highly branched dendritic trees into the OVLT beyond the BBB [26]. This finding suggests that GnRH dendrites and cell bodies residing in the OVLT itself and approximately within 100 μm from the OVLT in the deeper parenchyma can be exposed to molecules in the peripheral circulation even without the disruption of BBB.

The formation and integrity of the BBB and the infiltration of leukocytes to the CNS parenchyma is critically regulated by astrocytes [27,28,29]. It has been reported that astrocytes play both harmful and protective roles in neuroinflammation [30,31]. The physical barriers composed of the BBB and astrocytic end feet (glia limitans) around small vessels provide the first line of defense of the CNS against immune attacks [32]. Astrocytes are capable of inhibiting inflammation by maintaining the expression of junction proteins thereby preserving the intact BBB [33]. However, astrocytes may also be responsible for the expansion of CNS inflammation. In the presence of immune stimuli, astrocytes lose their end feet leading to the damage of glia limitans and also secrete factors, which are contributing to disruption of BBB [34]. Astrocytes also produce chemoattractant molecules that induce leukocyte infiltration [35].

Astrocytes-derived factors can also activate microglia, while vice versa microglia secrete factors that regulate the functions of astrocytes. The microglia–astrocyte crosstalk determines neuronal functions and dysfunctions [36]. Microglia are resident macrophages supplying essential defense of the CNS. Ramified resting microglial cells keep their environment under surveillance and they are immensely sensitive to the smallest disturbances in the extracellular environment responding rapidly with morphological and consequent functional changes. In response to stressors microglia are activated by gradually retracting their processes and becoming amoeboid phagocytic cells. Activated microglia phenotypes exist in a large scale spectrum with two end-points [37]. For instance, M1 phenotype microglia are pro-inflammatory involved in pathogen elimination, tissue damage, and neuroinflammation, while M2-like microglia are anti-inflammatory taking part in the resolution of inflammation and restoration of homeostasis [38].

## 3. Effects of Inflammation on Fertility: LPS-Induced Functional Disturbances in GnRH Neurons

There is numerous clinical evidence that infections and inflammatory diseases often impair reproductive functions in females [39,40,41,42]. It is well known that immune-stress can lead to reproductive dysfunction, such as anovulation and amenorrhea [43]. The suppressive effect of inflammation on the HPG axis is also well-documented in animal experiments. Most studies use peripheral injection of bacterial endotoxin, lipopolysaccharide (LPS), the major molecular component of the outer membrane of Gram-negative bacteria as a model to examine the impact of inflammation on the reproductive functions as LPS initiates an inflammatory response [44].

Both acute and prolonged inflammation induced by single or repeated peripheral injection of LPS inhibit secretion of GnRH and LH [45,46,47], while endotoxin can also disturb the ovarian responsiveness to gonadotropin stimulation [48,49]. Intravenous LPS administration decreases GnRH and GnRH receptor (GnRHR) mRNAs levels in the POA and ME in anestrous ewe [50]. Endotoxin injection also downregulates GnRHR gene expression in the OVLT and mPOA during the proestrous phase in female rats [51]. In the AP LPS injection reduces the sensitivity of cells to GnRH by downregulating GnRHR gene expression [50,51] and decreasing the gene expression of the β subunits of LH [52]. LPS injection suppresses tonic LH secretion and delays or prohibits the preovulatory LH surge in different species including rats [53], sheep [54], and nonhuman primates [55]. LPS itself may affect GnRH/LH secretion directly by binding to Toll-like receptors (TLR2/4) [56,57], which are found both in the hypothalamus and pituitary and indirectly by many factors like prostaglandins and opioids [49].

Importantly, the response to LPS is dose-dependent [58]. A high dose of LPS induces systemic inflammation such as sepsis resulting in multi-organ failure and death [59]. In contrast, a lower dose of LPS elicits a mild immune response without causing sepsis. However, limited number of studies examine the impact of mild inflammation on reproductive functions. Low single dose of LPS (500 ng/kg) from Salmonella Enteriditis, for instance, has been shown to dysregulate the expression of GnRH peptide in juvenile female pigs. This subclinical dose of LPS has increased the level of GnRH in the medial basal hypothalamus, the lateral hypothalamic are, the mammillary bodies, the median eminence and in the ovary without any clinical symptoms [60]. This result demonstrates that even an asymptomatic infection can disrupt homeostasis and cause reproductive dysfunctions. Our recently published paper also illustrates that a less severe immune-challenge may alter the integrity of HPG axis [61]. In our experiments we selectively induced a T-cell-dependent B-cell response with fluorescein isothiocyanate/keyhole limpet hemocyanin (KLH-FITC) and presented that KLH-FITC elicits ERK1/2 phosphorylation via IL-10 in female GnRH neurons in vivo [61].

## 4. Mechanisms of LPS-Induced Anti-Gonadotropic Effect of Inflammation on the HPG Axis

The LPS-induced anti-gonadotropic effect of inflammation is primarily mediated by pro-inflammatory cytokines in the hypothalamus. Among pro-inflammatory cytokines, IL-1β is the most potent inhibitor of the GnRH-LH system, IL-1α and TNF-α are less effective, whereas the participation of IL-6 appears irrelevant [62,63,64]. IL-1β regulates LH release primarily through modulation of GnRH neuronal activity. IL-1β might be responsible for most of the effects of LPS as intracerebroventricular (i.c.v.) injection of IL-1β has been shown to decrease GnRH mRNA level in the POA and ME [64]. Centrally administered IL-1β also suppresses GnRH translation in the hypothalamus [64,65]. Furthermore, IL-1β inhibits LH release by suppressing GnRHR gene expression in the ME [64] and POA [65] and by decreasing LHβ mRNA level [64,66] acting directly on IL-1β receptors of the pituitary gland [46].

Inflammation might cause these effects via fine-tuning molecular events and the structure of GnRH neurons. A study postulates that LPS suppresses GnRH synthesis at the posttranscriptional level rather than at the transcriptional level. This theory is based on the observation that LPS robustly decreases GnRH gene expression in the ME in the follicular phase of the estrous cycle of ewe while it does not change GnRH gene expression in the hypothalamic regions containing perikarya of GnRH neurons [67]. This finding is consistent with the characteristics of GnRH gene transcription. The amount of GnRH mRNA in the cytoplasm is higher than in the nucleus of GnRH neurons, [68,69] consequently GnRH transcript continuously translocated from the nucleus to the cytoplasm. Therefore, the change in GnRH mRNA levels may arise from nuclear events such as transcription or cytoplasmic events like modification of mRNA stability [70]. Accordingly, it is possible that the LPS-induced decrease of GnRH mRNA in the ME is a result of the degradation of cytoplasmic GnRH [50].

Another mechanism of action of LPS may include the inhibition of GnRH secretion via blocking GnRH mRNA transport. The transport of the GnRH transcript to the nerve terminals in the ME requires the integrity and proper functioning of cytoskeletal elements. Increasing evidence suggests that inflammatory cytokines induce cytoskeleton rearrangements in various cells such as cardiomyocytes, intestinal epithelium, or breast cancer cells [71,72,73]. Cytoskeleton organization is also affected by cytokines in neurons. Proinflammatory cytokines disrupt normal actin dynamics in Alzheimer’s disease [74], while IL-1β impairs the dendritic spine plasticity—substantial for LTP consolidation and memory formation—in hippocampal neurons by altering actin dynamics [75]. Although, it is not examined yet in GnRH neurons, it is possible that inflammation inhibits GnRH transport via proinflammatory cytokines by impairing the cytoskeleton.

## 5. Direct Effects of Cytokines on GnRH Neurons

Based on the findings that a subpopulation of GnRH neurons and their fibers could directly sense inflammatory molecules [26] including cytokines action in circumventricular organs [76,77,78], cytokines might be able to modify the functions of GnRH neurons directly. Although GnRH neurons are ideally situated to integrate immune responses on reproduction, little if any attention has been given to inflammatory factors monitoring of GnRH neurons.

Microarray studies showed that receptors associated with the progression of immune responses are abundantly expressed in mouse GnRH neurons such as interleukin, prostaglandin, TNF-α and receptors [79]. More recently immunohistochemical studies have also justified that immunomodulators can have direct impact on GnRH neurons. The expression of proinflammatory cytokine receptor IL-18Rα and the anti-inflammatory cytokine receptor IL-10R have been demonstrated in a portion of GnRH neurons providing the possibility for cytokines to act directly on GnRH neurons [61,80]. IL-10, for instance, is one of the most important anti-inflammatory cytokines balancing the immune response in the brain. Clinical studies have indicated that IL-10 is substantial for normal pregnancy, fertility, and fecundity [81,82,83], while IL-10 deficiency is associated with pregnancy loss, preterm birth or preeclampsia [84]. Although clinical investigations have shown correlation between the levels of peripheral IL-10 and pregnancy outcome, our recently published paper suggests that IL-10 may directly alter the function of GnRH neurons. Notably, we have found that the estrous cycle is perturbed in IL-10 KO mice, indicating that the action of IL-10 on GnRH neurons might help the maintenance of the integrity of the estrous cycle in bacterial/viral infection [61].

## 6. Indirect Cytokine Actions on GnRH Neurons: The Role of Glial Cells

GnRH neurons receive robust glial inputs regulating GnRH neuronal activity and secretion. The perykaria of GnRH neurons are enveloped in astrocytes, while three dimensional reconstruction of confocal images has revealed that microglia are in the vicinity of GnRH neurons [85].

Although astrocytes and microglia are in an optimal position for mediating immune responses to GnRH neurons, as they directly interact with GnRH neurons, their role in translating the effects of inflammation on the function of GnRH neurons is poorly understood. Previous studies have shown that astrocytes release immune modulators such as prostaglandin E2 (PGE2) and transforming growth factor-beta (TGFβ) to increase GnRH neuron firing and GnRH secretion under physiological conditions [86,87], but it is unexplored whether astrocytes influence GnRH functions during inflammation.

Microglia also release various cytokines. M1 phenotype microglia express pro-inflammatory factors such as interleukin 1α/β (IL-1α/β), interleukin-6 (IL-6) and tumor necrosis factor α (TNF-α), while M2-like microglia produce high levels of anti-inflammatory markers like IL-10 [38]. It has also been shown that ramified, surveying microglia but not the GnRH neuron itself express COX-1, one of the rate-limiting enzymes for prostaglandin (PG) synthesis [88]. The anatomical relationship of COX-1 immunopositive microglia and GnRH neurons and the fact that PGs are among the immune mediators influencing the regulation of GnRH secretion [89], suggest that the effect of PG on GnRH release might be due to the intercellular communication between microglia and GnRH neurons and could be disturbed during inflammation. A recently published study has described an indirect cytokine effect on GnRH neurons in aging-associated hypothalamic inflammation. In early aging TNF-α produced by activated microglia has been shown to inhibit GnRH gene expression [90].

## 7. Kisspeptin and RFamide-Related Peptides Mediate Inflammation on GnRH Neurons

Recent data presented that the kisspeptin system is sensitive to inflammation. Systemic endotoxin injection (LPS) in female rat decreases KISS-1 mRNA expression in the hypothalamus that consequently suppresses LH [91,92]. Moreover, intravenous (i.v.) injection of kisspeptin reverses LPS-caused LH suppression [93]. Another study using primary cultures of human fetal hypothalamic (hfHypo) cells containing 80% of GnRH neurons investigated the effect of the pro-inflammatory cytokine, TNF-α on GnRH release. They have found that TNF-α reduces GnRH secretion via downregulating kisspeptin signaling [94]. It is worth noting that GnRH and kisspeptin expressing cells do not form separate neuronal populations in hfHypo cells, but are coexpressed, suggesting that inflammation affects GnRH neurons rather directly by modifying kisspeptin signaling in hfHypo cells [94].

Other experiments also revealed that acute LPS treatment severely affects the GnRH pulse generators, KNDy neurons. In ovary-intact ewe dynorphin immunoreactive neurons are most active 6–7 h before the LH surge, while kisspeptin and NKB neurons are maximally activated during the LH surge. This activation pattern is disturbed by LPS preventing kisspeptin and dynorphin-positive cell activation leading to a failure to evoke an LH surge [95].

Inflammation may inhibit GnRH secretion via alteration of the RFRP system as LPS injection has been demonstrated to elevate hypothalamic RFRP and GPR147 mRNA levels in rodents [91,92]. Since RFRPs modulate kisspeptin signaling, inflammation might also have an effect on GnRH pulse generation via the RFRP system.

## 8. The Estradiol Feedback on GnRH Neurons During Inflammation

In addition to its role as a feedback molecule on GnRH neurons, estradiol modifies the response to inflammation. As the varying level of estradiol during the estrous cycle is a key factor in regulating the secretion of GnRH neurons and estradiol is a potent immunomediator [96], it is not surprising that the effect of inflammation on GnRH neurons greatly depends on the circulating concentration of estradiol. Experiment performed in ovariectomized ewes showed that endotoxin delays the estradiol-induced LH surge [97]. Nevertheless, the LPS-induced LH surge delay is time-dependent in relation to the onset of the estradiol stimulus. LPS blocks the estradiol-induced LH surge when it is infused at the beginning of estradiol rise. In contrast, endotoxin has no effect on LH surge when it is administered at a later stage closer to the commence of the surge when an increased level of estradiol is no longer necessary [97]. Other experiments carried out in ewes have suggested that the impact of inflammation on the GnRH mRNA expression in the hypothalamus is influenced by the circulating level of estradiol. LPS might decrease GnRH content via different mechanisms depending on the circulating estradiol concentration. LPS-induced inflammation decreases the transcription of GnRH mRNA in the POA during the anestrous phase when estradiol concentration is low [50]. Contrarily, endotoxin has no effect on GnRH gene expression during the follicular phase characterized by higher estradiol level. The authors propose that the decrease in the GnRH content of the POA during the follicular phase might be due to a reduced GnRH translation [67]. Another explanation can be that endotoxin lowers plasma estradiol concentrations in the follicular phase for the time of LH surge delay thereby blocking the preovulatory estradiol rise [98].

The Role of Cholinergic Anti-Inflammatory Pathway

The cholinergic anti-inflammatory pathway is an anti-inflammatory function of the efferent vagus nerve that inhibits systemic and local inflammation [99]. As immune cells in the spleen express acetylcholine receptors, the cholinergic anti-inflammatory pathway can control cytokine secretion [67,100]. An in vitro study in human macrophage cultures indicated that ACh attenuates the endotoxin-induced release of pro-inflammatory cytokines [101]. Later, in vivo studies have reported that blocking of acetylcholine (ACh) degradation by acetylcholinesterase (AChE), the enzyme responsible for the degradation of ACh markedly attenuated IL-1β expression in mouse hippocampus [102] and LPS-induced IL-1β production in sheep hypothalalmus [66]. More recent studies proved that the cholinergic anti-inflammatory pathway also has a role in hindering the effect of LPS on GnRH/LH secretion [66,67]. Peripherally administered AChEs (Neostigmine and Donepezil) eliminated the LPS-induced effects on the GnRH/LH system in the follicular phase of ewe estrous cycle. AChEs entirely abolished or reduced GnRH synthesis in the hypothalamus, while prohibited the suppression of LHβ gene expression and LH release and diminished the inhibition of GnRH receptor expression in the AP [67]. As parasympathetic vagus efferents are activated much faster to systemic inflammation than humoral anti-inflammatory pathways, the activation of the cholinergic anti-inflammatory pathway may serve as an important mechanism to restrict the magnitude of immune responses [101].

## 9. The Neuroinflammatory Processes and Function of GnRH Neurons in Aging

Aging is a gradual and general deterioration of physiological functions that affects the HPG axis. GnRH gene expression is reduced with aging leading to decreased GnRH secretion and reproductive decline [103]. The mechanism that accounts for the development of aging is unknown. Beyond its basic role in growth, development, reproduction, and metabolism, the hypothalamus has a fundamental role in systemic aging and lifespan control [104].

Aging is characterized by increased levels of circulating cytokines, pro-inflammatory markers and changes in the immune system called immunosenescence [37,105]. Similarly, mRNA levels of several cytokines and immune regulators elevated in the hypothalamus of aging mice. At the molecular level age-related inflammatory changes in the hypothalamus has been shown to be mediated by NF-κB and its upstream IκB kinase-β (IKKβ). During early aging NF-κB is activated in microglia leading to an overproduction of TNF-α. This cytokine then stimulates NF-κB signaling in hypothalamic neuronal cells. Importantly, activation of IKKβ/NF-κB inhibits GnRH release causing aging-related hypothalamic GnRH decline [90]. Interestingly, GnRH therapy or the inhibition of inflammation by blocking the activation of IKKβ or NF-κB can attenuate age-related symptoms leading to improved lifespan [90,106].

## 10. Pathophysiological Consequences: Effect of Obesity-Induced Inflammation on Reproductive Functions

In the last four decades, the prevalence of obesity and consequent reproductive problems have approximately tripled worldwide [13]. Optimal fat mass is indispensable for normal gonadal functions in adults, and both undernutrition and overnutrition inhibit gonadotropin production [107]. Female adult obesity is linked to menstrual cycle irregularities, ovulatory dysfunction and higher risk for miscarriages [108]. In addition to reproductive dysfunctions, chronic, low-grade systemic inflammation is also a hallmark of obesity [109]. Obesity develops when the energy homeostasis is disrupted [110]: The food intake, the energy expenditure and the energy storage become unbalanced [111]. Research over the past several decades has provided insight into control mechanisms of hypothalamus in the energy balance [112,113]. However, it is not entirely clear how obesity causes hypogonadism. Among several proposed mechanisms, one is that fat-rich diet induces hypothalamic inflammation, which in turn impairs the hormonal and neuronal circuits including the HPG axis causing reproductive disorders [114]. The mechanism by which fat-rich diet induces hypothalamic inflammation and subsequent HPG axis dysregulation is beginning to unfold. Experiments performed in dietary-induced obesity (DIO) mice suggest that hypothalamus has a prominent role in obesity-induced gonadotropin hormone level alterations. Several reports propose that hypothalamic cytokine expression pattern may have a remarkable role in the development of obesity-induced impairment of fertility [114,115]. DBA/2J mice fed high-fat diet develop dietary-induced obesity (DIO) and shows a significantly reduced pregnancy rate. It has been demonstrated that infertility manifested in DIO female DBA/2J mice is due to a suppressed GnRH expression [116]. In contrast, C57Bl/6J female mice are resistant to DIO. They require a long exposure to high-fat diet to develop obesity and even though they appear to have lengthened estrous cycles, the levels of gonadotropin hormones are not changed [115]. Ovariectomized (OVX) C57Bl/6J female mice, on the other hand, become responsive to DIO but remain resistant to gonadotropin hormone changes suggesting a protective mechanism—other than ovarian estradiol—against obesity-induced fertility problems in these female mice. It has been revealed that C57Bl/6J DIO female mice have increased levels of anti-inflammatory cytokine IL-10 in the hypothalamus, which may be able to prevent the hypothalamic inflammatory response and a consequent HPG axis damage [115]. More recently a novel concept (GELDING—Gut Endotoxin Leading to a Decline IN Gonadal function—theory) has been proposed to provide an explanation for the development of obesity-related hypogonadism. This theory hypothesizes that high fat diet alters the gut microbiome that leads to the breakdown of the intestinal mucosal barrier and the passage of bacterial endotoxin from the gut into the blood stream [117]. As a consequence, endotoxin inhibits progesterone production in the ovary [118]. However, it is unknown whether bacterial endotoxin leaking through the gut has an effect on the GnRH neuron.

Importantly, consumption of fat-rich food triggers astrocytes and microglia to produce pro-inflammatory cytokines via the master inflammatory NF-κB signaling pathway [119,120] leading to hypothalamic inflammation. Mounting evidence suggest that long-chain saturated fatty acids (SFAs) activate glial cells to induce inflammation [121,122]. It has also been proposed that SFAs can bind to TLR4 on astrocytes, microglia and neurons as well to initiate inflammation [123,124,125]. However, the role of TLR4 in generating inflammation is controversial. It has been shown in human macrophages that TLR4 is not a receptor for SFAs but alters the membrane lipid composition, that is necessary for SFA-induced inflammation [126].

The role of satiety molecules such as leptin and insulin is also important in regulating the function of GnRH neurons [127,128,129,130,131]. These neuropeptides control reproductive functions via modulation of GnRH neurons depending on the nutritional status [132]. Leptin is a hormone mainly produced by white adipose tissue that increases energy expenditure by activating catabolic and blocking anabolic neural circuits [133]. In addition, leptin triggers the expression of GnRH and the neural activity of GnRH neurons to secrete gonadotropin hormones [134,135]. Humans and mice lacking leptin (ob/ob mice) or leptin receptor (db/db mice) become obese and infertile [136]. As inflammation induces central leptin resistance, leptin is an important link between obesity and HPG axis defects [137]. Interestingly, serum leptin levels are positively correlated with insulin resistance (IR) [138] raising the possibility that leptin is also involved in regulating IR. Indeed, leptin regulates insulin receptor substrate-1 and 2 (IRS-1, IRS2) [139], modulates glucose metabolism and the function of insulin producing pancreatic β-cells [140]. Another essential metabolic factor involved in the impairment of GnRH function by obesity-associated inflammation is insulin signaling. Obesity-induces chronic low-grade inflammation is responsible for the progression of insulin resistance and accompanying type 2 diabetes and metabolic syndrome [141]. Cytokines derived from adipocytes, inflammasomes or activated macrophages and inflammatory signaling pathways link inflammation to IR [141]. Inflammatory cytokines such as TNF-α and IL-6 increase the phosphorylation of insulin receptor substrate-1 and/or 2 (IRS-1/2) via JNK, NF-κB, TLR4, and/or JAK-STAT signaling pathways that may inhibit insulin signaling finally leading to IR. The activation of JNK and NF-κB is also engaged in the generation of pro-inflammatory cytokines, which may in turn stimulate the pathways [141]. Subsequently, IR may perturb the HPG function as it has been published in mouse: brain-specific deletion of the insulin receptor results in hypogonadotropic hypogonadism [142]. It has also been demonstrated that insulin stimulates the secretion of GnRH [143]. In summary, inflammatory signals can alter the functions of GnRH neurons via reducing insulin related mechanisms.

Currently, it is not known how main metabolic peptides, including insulin and leptin influence the function of GnRH neurons as they are lacking the corresponding receptors. One hypothesis is that kisspeptin neurons are the central sensors for leptin and insulin, integrating and transmitting the metabolic signals to the GnRH neurons [135,144]. This theory is based on findings that kisspeptin neurons express leptin and insulin receptors [144,145,146,147]. Chronically obese female mice showed a decreased KISS-1 mRNA expression in the arcuate nucleus [148], whereas fasting also had a reducing effect on KISS-1 mRNA expression in the hypothalamus of female rats [149]. Diabetic female rats exhibited lowered KISS-1 mRNA levels in the hypothalamus [150]. Additionally, leptin elevates kisspeptin gene expression [151] and is able to depolarize kisspeptin neurons [152].

Interestingly studies investigating the association between obesity and estradiol levels are inconsistent in their findings [153,154,155]. A recently published report suggested a possible mechanism for how estradiol affects obesity [156]. Obesity is characterized by a pro-inflammatory state and accompanied by fertility problems. Estradiol can be a potential link between these anomalies as it is an effective anti-inflammatory factor and exerts negative feedback on gonadotropin secretion. Clinical studies comparing regularly menstruating obese and normal weight women have found that mean serum LH and its amplitude was significantly lower in obese women, while its pulse frequency was not changed suggesting the importance of pituitary in the observed alterations [156]. In addition, obese women had undoubtedly higher baseline pro-inflammatory cytokine levels such as IL-6 and IL-12. Following transdermal estrogen treatment mean LH and LH pulse amplitude increased in obese but decreased in normal weight participants [156]. Besides, estradiol treatment significantly decreased the levels of IL-1β, IL-12, and IL-8 in the serum obese subjects. FSH response was different between the two experimental groups (obese versus normal) when estradiol-treated participants received a physiologic i.v. GnRH bolus. In this case mean FSH decreased in normal weight but increased in obese women. These results provide evidence that exogenous E2 priming might have a beneficial effect on HPG axis function by improving gonadotrope sensitivity and chronic, systemic inflammation in ovulatory, obese women [156].

Taken together these findings suggest that attenuating chronic inflammation may ease the burden of obesity on fertility.

## 11. Conclusions

As discussed in this review inflammation is one of the underlying mechanisms of many pathological conditions such as bacterial/viral infections or obesity and even physiological processes such as aging. Inflammation may cause reproductive dysfunctions like infertility, subfertility and menstrual irregularities in all these conditions. As we pointed out the function of GnRH neurons is modified during inflammation. However, it is not clear how different pathologies alter the GnRH system. Gaining more information about the mechanism of inflammation-induced changes in the function of GnRH neurons may provide a solid platform for future therapies of heterogeneous fertility problems.
